# Antioxidant and Prooxidant Effects of Metal Dafonates on the NADPH-Dependent Lipid Peroxidation in the Murine Hepatic Microsomal System

**DOI:** 10.1155/MBD.1999.169

**Published:** 1999

**Authors:** M. Gupta, R. K. Kale, P. P. Kulkarni, S. B. Padhye

**Affiliations:** 1 Free Radical Biology Laboratory School of Life Sciences Jawaharlal Nehru University New Delhi 110 067 India; 2 Department of Chemistry University of Pune Pune 411 007 India

## Abstract

Dafone inhibits the lipid peroxidation significantly in a concentration dependent manner. The inhibition was found to be an uncompetitive type with the inhibition constant (Ki) of 62.5μM On the other hand complexation with metal ions results in a significant reversal from antioxidant to pro-oxidant properties for the resulting complexes which are cationic and with associated halometallate anions. The nature of the potentiation in case of the ferric compound was of competitive type with activation constant (Ka) having the value 32.5μM. The neutral copper-dafonate complex, however, inhibits lipid peroxidation with increase in concentration.


### ANTIOXIDANT AND PROOXIDANT EFFECTS OF METAL DAFONATES ON THE NADPH-DEPENDENT LIPID PEROXIDATION IN THE MURINE HEPATIC MICROSOMAL SYSTEM

M. Gupta[1], R. K. Kale*[1], P. P. Kulkarni[2] and S. B. Padhye*[2]

[1] Free Radical Biology Laboratory, School of Life Sciences, Jawaharlal Nehru University, New Delhi - 110 067, India
[2] Department of Chemistry, University of Pune, Pune - 411 007, India

**Abstract**

Dafone inhibits the lipid peroxidation significantly in a concentration dependent manner. The inhibition was found to be an uncompetitive type with the inhibition constant (Ki) of 62.5μM. On the other hand complexation with metal ions results in a significant reversal from antioxidant to pro-oxidant properties for the resulting complexes which are cationic and with associated halometallate anions. The nature of the potentiation in case of the ferric compound was of competitive type with activation constant (Ka) having the value 32.5 μM. The neutral copper-dafonate complex, however, inhibits lipid peroxidation with increase in concentration.

**Introduction**

1,10 Phenanthroline (phen, **I**) is a well-known metal complexing ligand yielding cationic or neutral complexes. The DNA cleaving activities of copper-phenanthroline compounds have been well documented[1]. 4,5 Diazafluoren-9-one (dafone, **II**), which is a prepared from 1,10 phenanthroline, bearing an exocyclic ketone function also undergoes complexation reactions with transition metal ions[2]. However, due to this exocyclic ketone in rigid five membered ring, the coordinating nitrogens are separated by considerable distance(2.99°A) compared to the distance in 1,10 phenanthroline ligand(2.66°A), which makes dafone some what difficult to coordinate with metal ions.

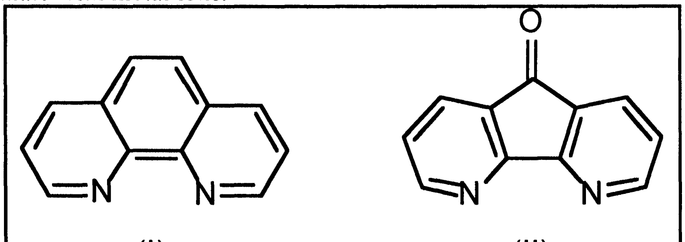

(I) (II)

Ruthenium complexes of dafone have been studied for their interactions with DNA[3]. However, the intracellular targets of these compounds and their resulting products still remain a matter of investigation. In the present work we have examined the effects of dafone and some of its metal complexes on the lipid peroxidation in the microsomes prepared from liver of mice. Peroxidation of membranes is known to induce a cascade of signal transduction leading to cell damage and death.

**Materials and Methods**

Chemicals:

Thiobarbituric acid (TBA) and NADPH were purchased from Sigma Co., USA. ADP and $FeCl_3$ for assays were obtained from Sisco, India. Dafone was prepared from 1,10-phenanthroline using literature procedure[4].

Synthesis:

All metal complexes were prepared by following a generalized procedure. The respective metal chlorides were mixed with the dafone ligand in a concentrated hydrochloric acid medium and stirred for half an hour. The corresponding metal complexes separated out as high quality polycrystalline material after a slow evaporation of the reaction mixture at room temperature. The detailed characterization and crystal structures of the compounds are reported elsewhere[5].

Animals:

Male Swiss albino mice (7-8 weeks old) maintained in the air-conditioned university animal facility were used. They were provided standard food (Hindustan Lever Ltd.) and water *ad libitum*. The studies were conducted according to ethical guidelines of the Indian National Science Academy (INSA) on the use of animals for scientific research.

Preparation of Microsomes:

Microsomes were prepared by the method described by Varshney and Kale[6] that involved sacrificing the mice by cervical dislocation and removing their livers immediately and chilling them in ice. The livers were finely minced and homogenized in a Potter- Elvejam homogenizer in 10 volume of 10mM Tris-HCl buffer (pH 7.4) containing 0.15M KCl. The suspension was centrifuged at 15,000 g for 30 min and the pellet was discarded. The supernatant was centrifuged at 105,000 g for 1 hr in XL-90 Beckman Ultracentrifuge. The pellet was dissolved in Tris-HCl buffer (pH 7.4) and the homogeneous supernatant microsomes were used in all further experiments for determination of lipid peroxidation. The freshly prepared microsomes by above method were used throughout the study.

Estimation of Lipid Peroxidation:

The assay system for NADPH-induced lipid peroxidation(2 ml) contained 0.6 mg proteins of microsomes, 50 mM $FeCl_3$, 4 mM ADP, 1 mM $KH_2PO_4$ and NADPH (0-25 μM) in 10 ml Tris-HCl buffer(pH 7.4) with and without dafone (50 μM-250 μM). Since the extent of lipid peroxidation was found to be highest at 45 min incubation time interval, it was used for the kinetic studies.

Lipid peroxidation was determined by TBA method and expressed in terms of malondialdehyde (MDA) formed per mg of protein. A typical assay consisted of following: 2 ml of assay solution was mixed with 0.5 ml of 30% TCA followed by 0.5 ml of 52 mM thiobarbituric acid and was heated for 30 min at 80°C. The solution was allowed to cool and then centrifuged at 6,000 rpm for 20 min at room temperature in Remi table-top centrifuge. The absorbance of the clear supernatant was measured at 531 nm on UV-2000 Hitachi spectrophotometer.

Protein Determination:

Protein concentration was determined by the method of Lowry et al.[7] using bovine serum albumin as the standard.

Determination of Apparent Michaelis-Menten constant (Km) :

The Lineweaver-Burk[8] plots were used to measure the Km value for lipid peroxidation determined under optimum conditions at 37(C using at least five concentrations of NADPH (5 to 25 μM). The results of the reciprocal velocity (1/v) expressed as nM malondialdehyde (MDA)/mg protein/min generated by a suitable amount of microsomes (0.6 mg) were plotted against the reciprocal of NADPH concentrations (1/s). The values of 1/Km and 1/Vmax were obtained from the intercepts on the x and y axes as usual.

Determination of Apparent Inhibition Constant (Ki) and Activation Constant (Ka) :

Inhibition constant (Ki) was determined by re-plotting the slopes of the Lineweaver-Burk plots against the dafone concentrations[9] which were linear yielding an intercept on the X-axis. The corresponding intercept on the Y-axis was a measure of Ki. Apparent activation constant (Ka) was also calculated by re-plotting the slopes of the Lineweaver-Burk plot against the reciprocal of the dafone concentrations.

### RESULTS

Synthesis

The crystal structure of the iron(III) compound, $[(dafone)_2H]^+[FeCl_4]^-$, shows a tetrachloro ferrite anion interacting with a proton shared in between two dafone molecules through H-bonding[5] while nickel(II) compound is formulated as $[(dafoneH)_2]^{2+}[NiCl_4]^{2-}$. The copper-dafonate was obtained as a blue-green crystalline material having a composition $Cu(dafone)_2Cl_2$ similar to that reported by Rajasekharan et al.[2a]. In this compound two dafone ligands are bound to copper as neutral species in the equatorial plane while the two chlorides are bonded at axial positions yielding essentially an octahedral complex.

Time course of lipid peroxidation

In order to determine the time course of lipid peroxidation, two ml of microsomes (0.6 mg proteins/ml) were incubated in the presence and absence of NADPH for different periods (0 to 120 min) of time. NADPH-dependent lipid peroxidation increases with increase in the incubation time (**Fig.1**). The levels of spontaneous or uncatalyzed lipid peroxidation of microsomes are very low compared to the NADPH catalyzed lipid peroxidation. The extent of NADPH-induced lipid peroxidation is highest at 45 min incubation time intervals which is used for further studies.

Effect of dafone on lipid peroxidation

The effect of various concentrations of dafone on NADPH-induced microsomal lipid peroxidation is shown in (**Fig.2a**). It is evident that dafone (50-150 μM) inhibits the lipid peroxidation in a concentration-dependent manner (**Fig.2b**).

Kinetics of lipid peroxidation

The Lineweaveer-Burk plots for NADPH induced lipid peroxidation in microsomes are perfectly linear indicating that the Michaelis-Menten relation is applicable. These plots measured at different concentrations of dafone (50 to 150 μM) showed uncompetitive inhibition (**Fig.3a**). The Km values vary depending upon the concentration of dafone (**Table 1**). The inhibition constant, (Ki) is obtained by re-plotting the slopes of Lineweaver-Burk plots against dafone concentrations (**Fig.3b**) and is found to be 62.5 μM.

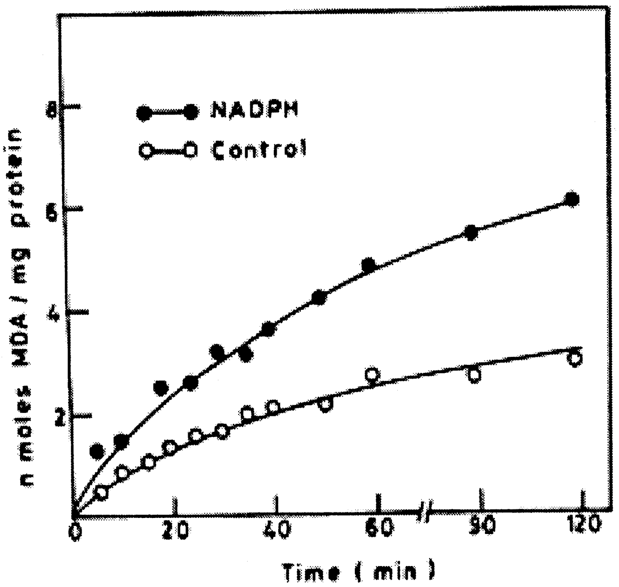

**Figure 1. NADPH-induced lipid peroxidation.**

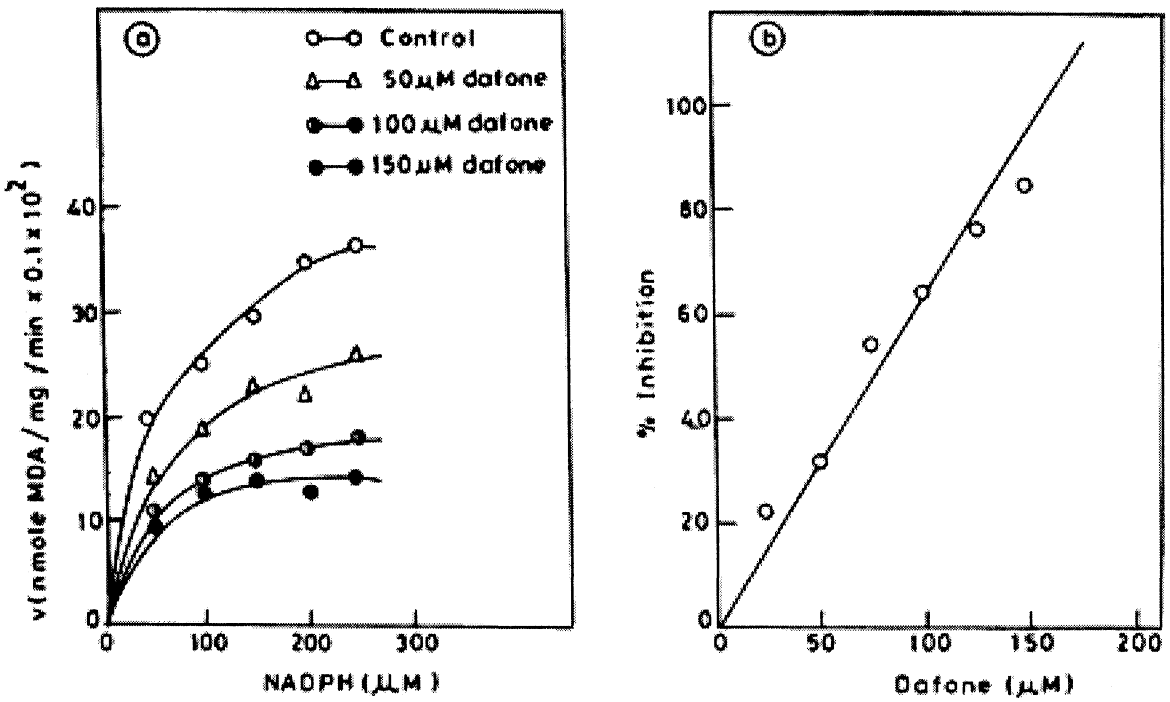

**Figure 2. Effect of different concentration of dafone on Lipid peroxidation.**

Effect of metal complexes of dafone on lipid peroxidation

The effect of metal-dafonates on the NADPH-induced lipid peroxidation is shown in **Fig.4**. Two ml of microsomes (0.6 mg proteins/ml) were incubated with or without metal complexes (100 μM) for different (0 to 120 min) time intervals. The Fe-dafonate and Ni-dafonate complexes both show increased lipid peroxidation with the former showing a more pronounced effect. The enhancement is proportional to the incubation time used. On the other hand, the copper-dafonate compound shows no potentiation of lipid peroxidation. The levels of lipid peroxidation are lower than control. However, it had a higher value of lipid peroxidation than dafone itself. The concentration dependence studies show the same trends for all the compounds (**Table 2**). It is thus clear that complexation of dafone with Fe and Ni ions changes its ability from being antioxidant to pro-oxidant compound towards NADPH-dependent lipid peroxidation in rat liver microsomes. Since the Fe-dafonate compound showed a marked increase in lipid peroxidation, the kinetics of its effect on NADPH-induced lipid peroxidation was studied in detail.

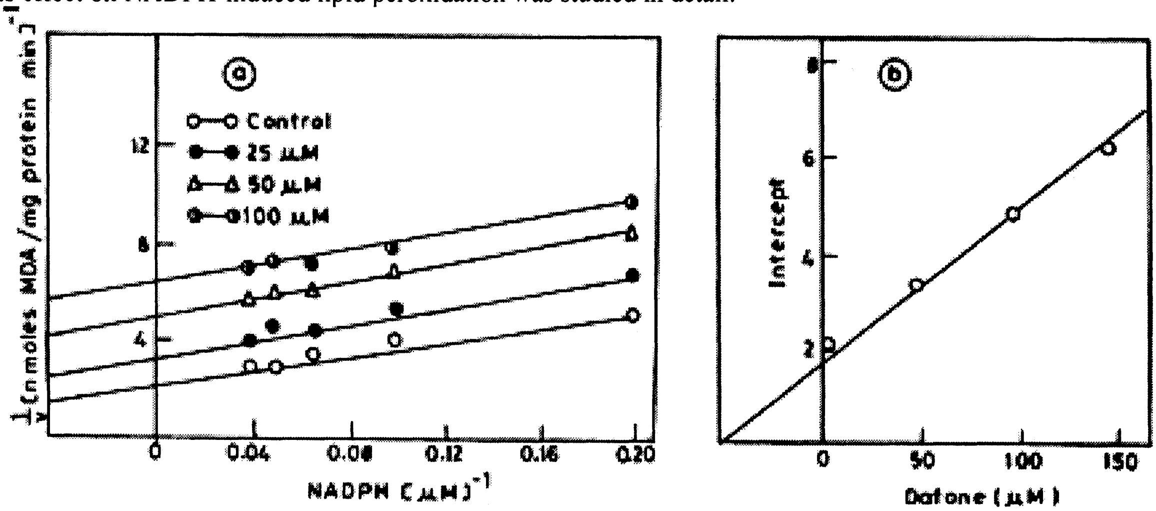

**Figure 3. Kinetics of lipid peroxidation by dafone.**

| Compound | Concentration (µM) | | | |
|---|---|---|---|---|
| | 0 | 25 | 50 | 75 |
| | Km | | | |
| Dafone | 7.1(0.63) | 5.5(0.49) | 3.8(0.12) | 2.7(0.09) |
| Fe-dafone | 25(1.12) | 15.3(0.08) | 10.0(0.62) | 5.6(0.032) |
| | Vmax | | | |
| Dafone | 0.79(0.08) | 0.5(0.043) | 0.34(0.052) | 0.26(0.021) |
| Fe-dafone | 0.58(0.032) | 0.58(0.029) | 0.58(0.042) | 0.58(0.025) |

**Table 1. Effect of different concentrations of dafone and Fe-dafone compound on Km and Vmax. Values.**

Microsomes (0.6 mg proteins) were incubated under optimum conditions for 45 min. Lipid peroxidation was determined from MDA formation. Km (µM NADPH) were obtained by the Lineweaver-Burk plots in the absence of additives. Each value represents mean of at least four experiments.

Kinetics of Lipid peroxidation by Fe-dafonate

The Lineweaver-Burk plots for the NADPH-induced lipid peroxidation with the Fe-dafonate complex (50 to 150 µM) showed a competitive type of activation (**Fig.5a**) and the Km values were found to be varying with concentrations of the Fe-dafonate complex (**Table 1**).

**Discussion**

Phenanthroline-type ligands have been extensively studied for the DNA binding activities of their ruthenium compounds[3]. When functionalized with a methylene bridge bearing keto group as in dafone(**II**), the DNA binding characteristics of the resulting compound are expected to be modified considerably due to the distortion caused in the bite distances of the coordinating nitrogens. Such modified DNA binding patterns can result in altered biological response. However, it may be mentioned that biologically active compounds are known to have multiple mechanisms of action. The same may be true for dafone and its metal conjugates. Fragmentation of nuclear DNA into oligonucleosomal subunit is one of the characteristics of apoptosis, a distinct mode of cell death that plays significant role in many different aspects of biology and medicine[10]. Since membrane lipid peroxidation is considered to be a critical event in the series of steps that leads to apoptotic cell death[11], modulation of lipid peroxidation by dafone and its metal conjugates might also result in altered biological effect in terms of cellular damage and death.

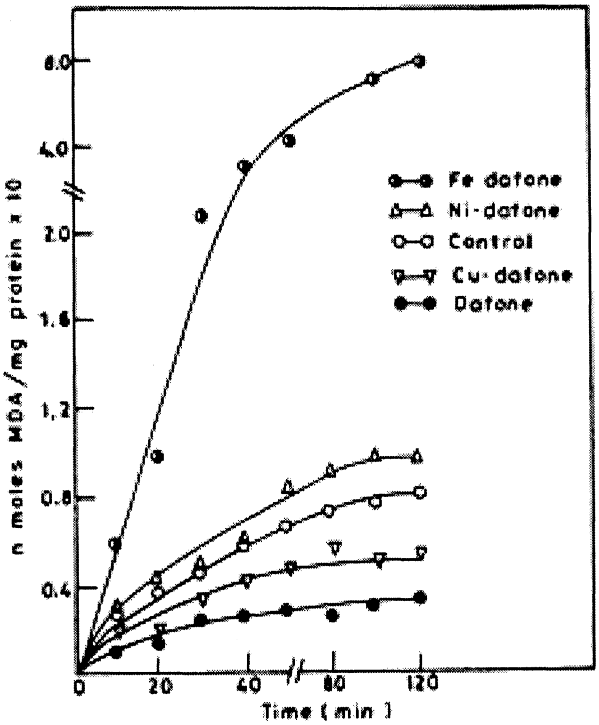

**Figure 4. Effect of metal-conjugates on lipid peroxidation.**

| Concentration | Additives | | | |
|---|---|---|---|---|
| μM | Dafone | Cu-dafone | Ni-dafone | Fe-dafone |
| 0 | 0.25( 0.05) | 0.24(0.03) | 0.22(0.06) | 0.25(0.015) |
| 50 | 0.18(0.021) | 0.22(0.026) | 0.24(0.019) | 0.36(0.031) |
| 100 | 0.16(0.014) | 0.16(0.012) | 0.29(0.025) | 0.49(0.036) |
| 150 | 0.10(0.01) | 0.14(0.011) | 0.33(0.031) | 0.63(0.05) |
| 200 | 0.083(0.09) | 0.11(0.01) | 0.37(0.02) | 0.85(0.15) |
| 250 | 0.052(0.07) | 0.08(0.042) | 0.41(0.035) | 1.1(0.11) |

**Table 2. Effect of various concentrations of dafone and dafone compounds on NADPH induced lipid peroxidation (MDA/mg) in 0.6 mg microsomes.**
Values are mean ± S.D.> of at least four experiments.

The present study indicates that the appended ligand, viz. dafone(**II**) shows very low levels of electrostatic association with the substrate. However, in the presence of halometallate anions accompanying the protonated cationic form of the ligand, an enhanced lipid peroxidation is observed. The NADPH-dependent microsomal lipid peroxidation is known to be promoted by an iron-oxygen complex[12]. The activated complex is generated by providing a reducing equivalent to the system containing ADP-$Fe^{3+}$ and $O_2$ by the NADPH-cytochrome P450 reductase which abstracts the allylic hydrogen from the polyunsaturated fatty acids (LH) (e.g. reactions 1 to 3) which leads to the initiation of lipid peroxidation.

$$\text{NADPH} + \text{ADP-Fe}^{3+} \rightarrow \text{NADP}^{+} + \text{ADP-Fe}^{2+} + \text{H}^{+} \quad (1)$$

$$\text{ADP-Fe}^{2+} + \text{O}_2 \rightarrow \text{ADP-Fe}^{2+}\text{-O}_2 \leftrightarrow \text{ADP-Fe}^{3+}\text{-O}_2^{-} \quad (2)$$

$$\text{LH} + \text{ADP-Fe}^{3+}\text{-O}_2^{-} \rightarrow \text{L.} + \text{ADP-Fe}^{2+}\text{-O}_2\text{H} \quad (3)$$

Our studies indicate that the cationic dafone dimer stabilized through H-bonding in the presence of $[Fe(III)Cl_4]^-$ anion is found to enhance the lipid peroxidation in a concentration dependent manner. It has been established that complexing agents promote protein damages and lipid peroxidation in the presence of transition metal ions probably through alteration of the redox potentials of the ligand[13]. Enhanced lipid peroxidation particularly by iron compound of dafone is likely to trigger biochemical events that can cause a

DNA fragmentation and in turn cellular damage or apoptotic cell death. The electrostatic association with DNA may also be promoted through the cationic dafone conjugates making it susceptible for fragmentation and contributing to an oxidative damage and apoptotic cell death.

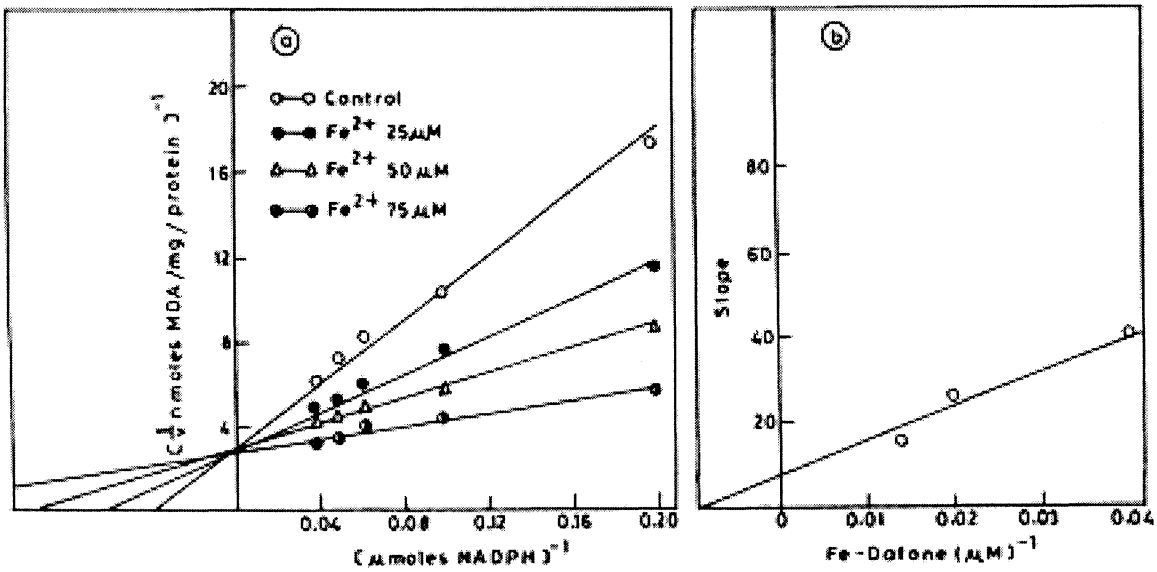

**Figure 5. Kinetics of lipid peroxidation by Fe-dafonate.**

Lineweaver-Burk plots with different concentrations of Fe-dafone compound reveal a competitive type of activation (**Fig 4**) which suggests that the cationic dimeric form of **II** stabilized through H-bonding by the tetrahalometallate anion is an important electrostatic component in the binding of proteins and the subsequent degradation. In order to confirm the validity of such a possibility, we included a neutral, monomeric copper complex of dafone reported by Rajsekharan et al[3a]. in the present study. As anticipated, it showed negligible amount of lipid peroxidation. Thus, results of present work indicate that parent dafone ligand can prevent the oxidative damage while its cationic metal conjugates with associated halometalate anions can potentiate it. Since these properties can be varied in a pre-designed manner dafone compounds are potentially useful compounds for the systematic studies of DNA cleavages and oxidative damages as well as their possible link through signal transduction. Results of such studies can provide crucial inputs for developing new metal-based drugs.

**Acknowledgements**

MG and PPK acknowledge financial assistance from UGC and CSIR (New Delhi, INDIA) respectively while SBP would like to thank British Council for the Link Program and Professor Ekk Sinn, University of Hull (UK) for the structural studies and wonderful hospitality.

**References**

1. Sigman D.S., Graham D.R., D'Aurora V. and Stern A.M.; *J. Biol. Chem.* **254** No.24, 12269(1979).
2. (a) Balagopalakrishna C., Rajasekharan M.V., Bott S., Atwood J.L. and Ramakrishna B.L.; *Inorg. Chem.* **31**,2843(1992); (b) Lu Z., Duan C., Tain Y., You X. and Huang X. *Inorg. Chem.* **35**, 2253(1996).
3. Pyle A.M., Rahman J.P., Meshoyrer R., Kumar C.V., Turro N.J. and Barton J.K.; *J. Am. Chem. Soc.* **111**, 3051(1989).
4. Henderson L.J. Jr., Fronczek F.R. and Cherry W.R.; *J. Am. Chem. Soc.* **106** 5876(1984).
5. Part of the work has been presented as an Abstract at *214th ACS National Meeting at Las Vegas* in Sept. 1997, Paper No. 90. Kulkarni P., Padhye S. and Sinn E.
6. Varshney R. and Kale R.K. *Int. J. Radiat. Biol.* **58** 733(1990).
7. Lowry O.H., Rosenbrough N.J., Farr A.L. and Randall *J. Biol. Chem.* **193**, 265(1951).
8. Lineweaver H. and Burk D. *J. Am. Chem. Soc.* **56**, 658(1934).
9. M. Dixon and E.C. Web (1979) in *Enzymes*, 4th Edition, pp. 82, Longman Publ.
10. Wyllie A.H., Kerr J.R.F. and Currie A.R. *Int. Rev. Cytol.* **68**, 251(1980); Williams G.T., Smith C.A., McCarthy N.J. and Grimes E.A. *Trends Cell Biol.* **2**, 263(1992); White E. *Genes Dev.* **10**, 1(1996).

11. Ramakrishnan N., McClain D.E. and Cantravas G.N. *Int. J. Radiat. Biol.* **63**, 693(1993); Ramkrishnan N., Kalinich J.F. and McClain D.E. *Biochem. Pharmacol.* **51**, 1443(1996).
12. Svigen B.A., Buege J.A., O'Neal F.O. and Aust S.D. *J. Biol. Chem.* **254**, 5892(1979).
13. Kumbhar A.S., PadhyeS.B., Jitender and Kale R.K.; *Metal-based Drugs* **4**, 279(1997).

**Received: April 21, 1999 - Accepted: May 11, 1999 - Received in revised camera-ready format: May 18, 1999**

